# Interleukin-18 does not influence infarct volume or functional outcome in the early stage after transient focal brain ischemia in mice

**DOI:** 10.1186/2040-7378-2-1

**Published:** 2010-01-05

**Authors:** Stefan Braeuninger, Christoph Kleinschnitz, Guido Stoll

**Affiliations:** 1 Department of Neurology, Julius-Maximilians-Universitaet Wuerzburg, Josef-Schneider-Strasse 11, D-97080 Wuerzburg, Germany

## Abstract

Interleukin-18 (IL-18) is a proinflammatory cytokine of the interleukin-1 family which is upregulated after cerebral ischemia. The functional role of IL-18 in cerebral ischemia is unknown. In the present study, we compared infarct size in IL-18 knock-out and wild-type mice 24 hours and 48 hours after 1-hour transient middle cerebral artery occlusion (tMCAO). Moreover, the functional outcome was evaluated in a modified Bederson score, foot fault test and grip test. There were no significant differences in infarct size or functional outcome tests between wild-type and IL-18 knock-out mice. These data indicate that the early inflammatory response to cerebral ischemia does not involve IL-18, in contrast to other interleukin-1 family members such as interleukin-1.

## Background

There is increasing evidence that inflammatory processes play a detrimental role in ischemic stroke [1,2]. On the other hand, the postischemic immune response may also be beneficial with respect to neuroprotection and tissue remodelling. The proinflammatory cytokine IL-18 is an interleukin-1 family member identified as and named interferon-γ-inducing factor [3,4]. The effects of IL-18 are complex and pleiotropic involving activation of T cells and NK cells in autoimmune disorders (for review, see Reddy [5]). In the central nervous system, IL-18 can locally be produced by activated microglia [6,7]. Increased IL-18 serum levels have been detected within 24 hours in patients with acute ischemic stroke [8], and elevated IL-18 plasma levels at 48 hours were associated with unfavorable clinical outcome at 3 months [9]. Moreover, in hypoxic-ischemic brain injury in neonatal rats, an early IL-18 activation (already within hours) and a progressive increase for at least 14 days have been described [10]. At 3 days after hypoxia-ischemia, IL-18 deficiency has been shown to ameliorate infarct volume and grey matter injury [10] as well as white matter injury [11] in neonatal mice. These studies suggest that IL-18 may play a pathophysiological role in stroke development. To elucidate a functional role of IL-18 in cerebral ischemia we investigated infarct size and functional outcome 24 hours and 48 hours after tMCAO in adult mice with IL-18 deficiency.

## Methods

Animal studies were conducted in accordance with institutional guidelines and approved by the appropriate authorities (Regierung von Unterfranken). Wild-type and IL-18 knock-out mice that had been generated [12] and backcrossed onto BALB/C mice [13] as described were kindly provided by Drs. X-Q Wei and FY Liew, Glasgow, Scotland, and bred and raised in our laboratory animal facility. A total of 35 wild-type and 29 IL-18 knock-out animals (plus additional 11 wild-type and 10 knock-out mice for laser-Doppler flowmetry and ink perfusion) were used.

Mice weighting 18-24 g were subjected to transient focal cerebral ischemia in the right middle cerebral artery (MCA) territory for 1 hour using the intraluminal suture MCA occlusion method [14]. In brief, mice were anesthetized with 2% to 2.5% enflurane in a 70% N_2_O/30% O_2 _mixture. A servo-controlled heating blanket was used to maintain core body temperature close to 37°C throughout surgery. A silicon rubber-coated 6.0 nylon monofilament (Doccol, Albuquerque, NM) was inserted into the right common carotid artery and advanced via the internal carotid artery to occlude the origin of the MCA, causing focal ischemic brain injury in the right MCA territory. The occluding filament was removed after 1 hour to allow reperfusion. Animals were sacrificed 24 hours or 48 hours after tMCAO. Brains were harvested and 2 mm-thick coronal slices were sectioned in a mouse brain matrix. After staining with 2% 2,3,5-triphenyltetrazolium chloride (TTC; Sigma-Aldrich, St. Louis, MO) in PBS, the pale infarctions were readily discernable from the brick-red non-ischemic areas and planimetric measurements were obtained using the ImageJ software package (available at http://rsb.info.nih.gov/ij/; National Institutes of Health, Bethesda, MD). The calculated lesion volume was corrected for brain swelling as described by Ginsberg et al. [15].

Additionally, we assessed the functional outcome in representative subsets of the animals. Immediately after recovery from anesthesia, and 24 hours and 48 hours later, a modified Bederson score [16] was determined according to the following scoring system: 0, no deficit; 1, forelimb flexion; 2, as for 1, plus decreased resistance to lateral push; 3, unidirectional circling; 4, longitudinal spinning or seizure activity; 5, no movement. 24 hours and 48 hours after surgery, the foot fault test and grip test were performed. The foot fault test was done as described by Gibson et al. [17], with the following modifications: Mice were placed on an elevated grid with 1.44 cm^2 ^openings and allowed to take 25 paired steps. Animals not moving spontaneously for at least 25 steps were excluded. The number of foot faults of the ipsilateral and contralateral limbs was counted. Foot faults were given as the percentage of contralateral (left) limb foot fault errors of all errors made. The grip test, also known as string test, was adopted from Moran et al. [18], with modified scoring system. For this test, the mouse was placed midway on a string between two supports and rated as follows: 0, falls off; 1, hangs onto string by one or both forepaws; 2, as for 1, and attempts to climb onto string; 3, hangs onto string by one or both forepaws plus one or both hindpaws; 4, hangs onto string by fore- and hindpaws plus tail wrapped around string; 5, escape (to the supports).

Laser-Doppler flowmetry (Moor Instruments, Axminster, United Kingdom) was used to monitor cerebral blood flow in 6 IL-18 +/+ and 5 IL-18 -/- animals before surgery (baseline), immediately after tMCAO, and 5 minutes after removal of the occluding monofilament (reperfusion). For this, a flexible laser-Doppler probe was positioned perpendicular to the exposed skull 2 mm posterior and 6 mm dexterolateral to the bregma, corresponding to the laser-Doppler probe position in murine MCA occlusion reported by Connolly et al. [19]. These animals were not included in the infarct size and functional outcome evaluations, because laser-Doppler flowmetric measurements in addition to tMCAO inevitably leads to prolonged operation times.

To exclude anatomic differences that could cause different susceptibility to tMCAO in wild-type and knock-out mice, we studied the cerebrovasculature in 5 IL-18 +/+ and 5 IL-18 -/- mice. Animals were deeply anesthetized with CO_2 _and transcardially perfused with 4% paraformaldehyde and then with black ink (T25; Edding, Ahrensburg, Germany). After brain removal and overnight fixation in 4% paraformaldehyde, the circle of Willis was visualized under a dissecting microscope. Special attention was paid to the posterior communicating arteries, whose level of plasticity was rated as described by Murakami et al. [20]: 0, absent; 1, capillary anastomosis; 2, small truncal vessel; 3, patent. A posterior communicating artery with a score of 0 or 1 is regarded as hypoplastic, and with a score of 2 or 3 as normal.

Corrected infarct volumes, data from the functional outcome tests and from the posterior communicating artery score, and laser-Doppler flow measurements were statistically analyzed in two-tailed Mann-Whitney U tests using Prism 4 (GraphPad Software, San Diego, CA).

## Results

**Figure 1:**
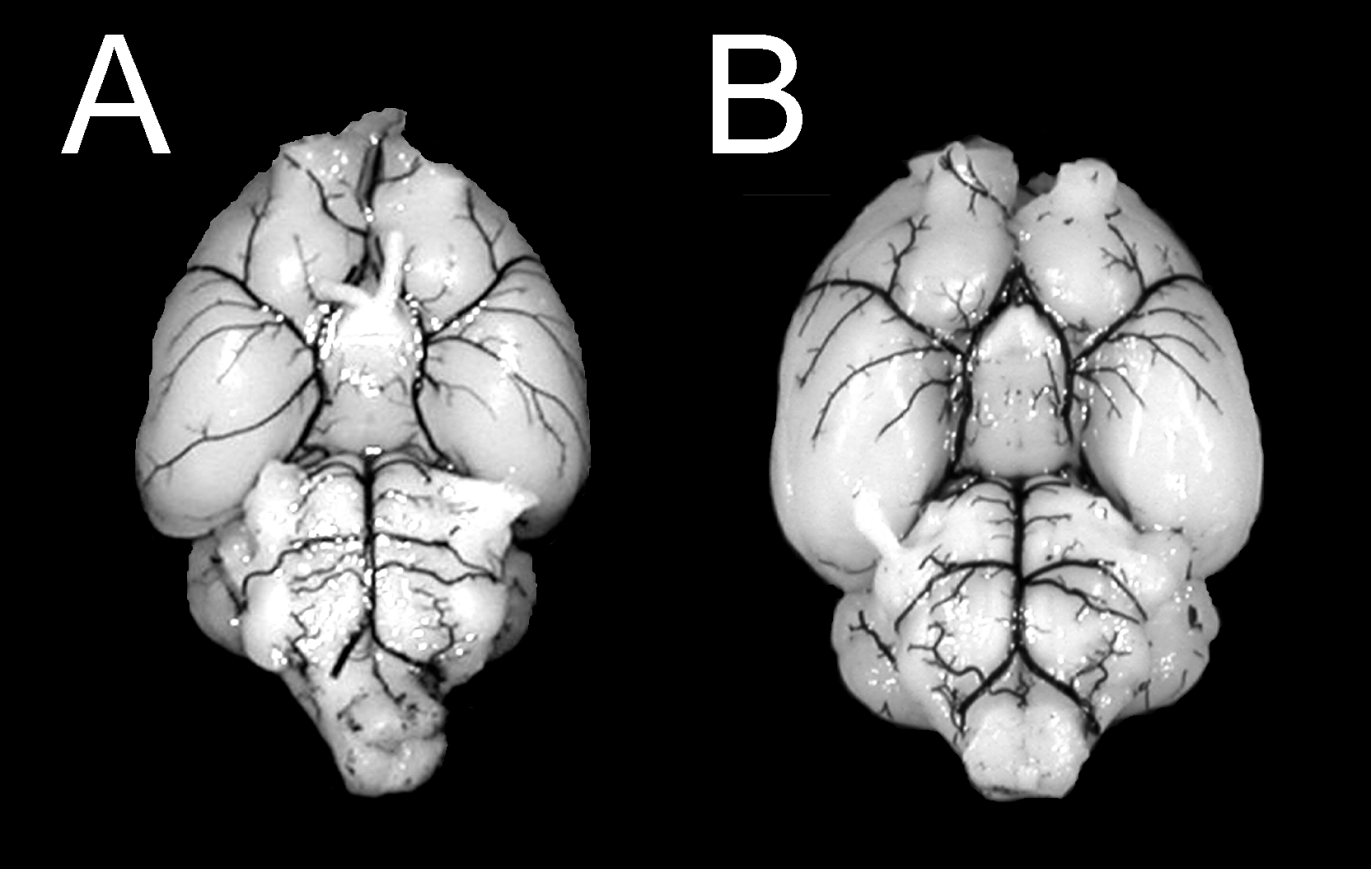
**Ink perfusion**. Images of a BALB/C wild-type mouse brain (A) and of a brain from an interleukin-18 knock-out mouse on a BALB/C background (B) with ink-perfused cerebrovasculature.

Ink perfusion was performed in IL-18 wild-type and knock-out mice to visualize the complete circle of Willis. No gross anatomic differences were noted that could influence stroke outcome (Figure 1). The score assessing formation of the posterior communicating arteries of both hemispheres, which are pivotal in collateral blood flow between the anterior and posterior circulation, did not differ significantly in wild-type and knock-out mice (median of 2 in both groups; p = 0.91). Moreover, Laser-Doppler flowmetry ensured technical accuracy and similar basic characteristics in IL-18 +/+ and IL-18 -/- mice, since it did not show any significant differences between wild-type and knock-out animals. After right MCA occlusion, there was a similar substantial reduction of right hemispheric cerebral blood flow (median, 16.15% and 16.4%, respectively; p = 0.93). The blood flow recovered to a median of approximately 50% of baseline blood flow already within minutes after removal of the occluding intraluminal monofilament (median, 48.9% and 50.9%, respectively; p = 1.00).

**Figure 2:**
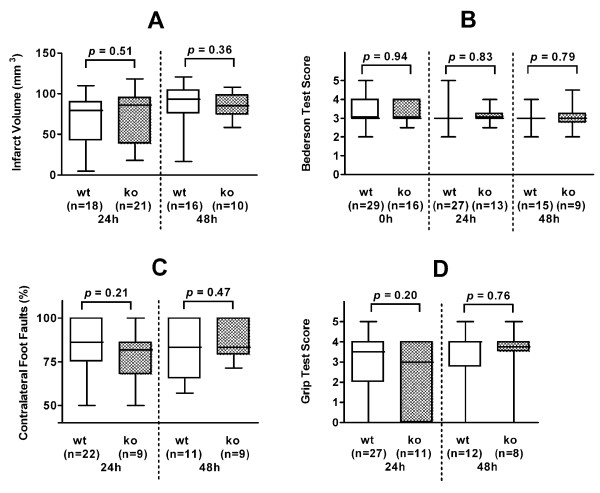
**Infarct volumes and functional outcome**. Infarct volumes (A) and functional outcome scores (B: modified Bederson test score; C: foot fault test; D: grip test score) of interleukin-18 wild-type and knock-out mice. The results are diagrammed as whisker boxes with medians. Boxes represent interquartile ranges and whiskers indicate extreme values. The p values resulting from Mann-Whitney U tests are given; all p values were greater than 0.05 and were thus considered insignificant. Abbreviations: wt, wild-type animals; ko, interleukin-18 knock-out animals; n, number of animals; 0 h, 0 hours (postoperatively after recovery from anesthesia); 24 h and 48 h, 24 hours and 48 hours after 1-hour middle cerebral artery occlusion.

We next assessed the influence of IL-18 on infarct size and on functional outcome. There were no significant differences in edema-corrected infarct size on standardized TTC-stained brain slices between wild-type and IL-18 knock-out mice after 24 hours (median, 79.25 mm3 and 86.1 mm^3^, respectively; interquartile range, 41.9 - 90.3 mm^3 ^and 38.1 - 95.6 mm^3^, respectively; p = 0.51) or 48 hours (median, 93.05 mm^3 ^and 85.2 mm^3^, respectively; interquartile range, 75.3 - 104.6 mm^3 ^and 73.9 - 98.7 mm^3^, respectively; p = 0.36). These results are presented in Figure 2A. The functional outcome scores were not significantly different in IL-18 +/+ as compared to IL-18 -/- mice, too (Fig. 2B, C, D).

## Discussion

As principal finding, we show that deficiency of IL-18 does not protect mice from ischemic brain damage after tMCAO. These findings are surprising given the reported upregulation of IL-18 blood levels in stroke patients [8,9] associated with adverse clinical outcome [9] and the profound impact of IL-18 in experimental neonatal stroke [10,11]. However, two recent nested case-control studies have not confirmed an association of IL-18 with increased risk of stroke in older people [21] or with recurrent stroke [22]. Moreover, our data are in accordance with a previous study by Wheeler et al. showing no differences in infarct size at 24 hours between wild-type and IL-18 -/- mice on a C57BL/6 background subjected to 15 and 30 minutes of tMCAO which leads to smaller infarcts than 1 hour occlusion time [23]. We extend this previous study by applying a longer tMCAO time (1 hour) leading to infarcts involving the entire MCA territory, by assessing functional outcome and by following infarct development up to 48 hours. Recently, IL-18 expression and activation has been described already at 24 hours in a thromboembolic murine stroke model [24]. In contrast, in the rat, IL-18 mRNA expression was increased later at 48 hours, and peaked between 7 and 14 days [25]. IL-18 has been localized to microglia/macrophages within ischemic lesions [25]. The structurally similar interleukin-1β reached a peak already within 16 hours and was rapidly downregulated subsequently [25]. Thus, unlike interleukin-1β, IL-18 seems to be associated with the mid-stage inflammatory response to ischemic brain lesions. Accordingly, similar findings were reported for traumatic brain injury in mice: IL-18 was significantly elevated at 7 days, but not within 4 hours to 24 hours, after experimental closed head injury as compared to sham treatment [26]. Inhibition of IL-18 by IL-18-binding protein resulted in improved neurological recovery by 7 days, while brain edema at 24 hours was not reduced [26]. The cytokine response in neonatal rodents subjected to hypoxic-ischemic brain injury may differ. Here, a significant mRNA elevation for IL-18 has been reported to occur already at 3 hours, but also progressively increased until day 14 [10]. In summary, our findings in adult IL-18 knock-out mice support the notion that IL-18 is not functionally relevant for early stroke development, but may play a role in late-stage neuroinflammation after stroke which awaits further elucidation.

## Competing interests

The authors declare that they have no competing interests.

## Authors' contributions

SB and CK wrote the paper and conducted the experiments. GS designed the study and reviewed the paper. All authors read and approved the final manuscript.
